# Supplementary material for: Development of a computer-aided design software for the quantitative evaluation of aesthetic damage
Source: PLoS One. 2019 Dec 18;14(12):e0226322. doi: 10.1371/journal.pone.0226322 (PMC6919621; doi:10.1371/journal.pone.0226322)
Supplement: S1 Code site — 1. (ZIP) [file pone.0226322.s001.zip › [EESC_jr][Projeto]Relatorio_07_21/reports/templates_zip/gentelella-master/vendors/jszip/documentation/_layouts/default.html]

{{page.title}}


**JS**Zip

- API
- How to / examples
- Performances / limitations

- JSZip on Github

{% if page.section and page.fullpage != true %}

{% case page.section %}
{% when "main" %}

#### JSZip users :

- Installation
- FAQ
- Changelog
- Upgrade guide
- Bug tracker

#### JSZip developers :

- How to contribute
- Contributors

{% when "api" %}

- JSZip
  - new JSZip() or JSZip()
  - new JSZip(data [,options])
  - JSZip#file(name)
  - JSZip#file(regex)
  - JSZip#file(name, data [,options])
  - JSZip#folder(name)
  - JSZip#folder(regex)
  - JSZip#filter(predicate)
  - JSZip#remove(name)
  - JSZip#generate(options)
  - JSZip#load(data [, options])
  - JSZip.support
- ZipObject

{% when "example" %}

#### How to ...

- Use JSZip
- Read a file
- Write a file

#### Examples

- Read local file
- Read remote file
- Give the user its zip file
- Mini app : downloader

{% endcase %}

{% endif %}

# {{page.title}}


{{content}}
